# Characterization of Different Types of Excitability in Large Somatosensory Neurons and Its Plastic Changes in Pathological Pain States

**DOI:** 10.3390/ijms19010161

**Published:** 2018-01-05

**Authors:** Rou-Gang Xie, Wen-Guang Chu, San-Jue Hu, Ceng Luo

**Affiliations:** Department of Neurobiology and Collaborative Innovation Center for Brain Science, Fourth Military Medical University, Xi’an 710032, China; rgxie@fmmu.edu.cn (R.-G.X.); chuweng@fmmu.edu.cn (W.-G.C.); sjhu@fmmu.edu.cn (S.-J.H.)

**Keywords:** excitability type, large DRG neurons, hyperpolarization-activated cation current, chronic compression of dorsal root ganglion

## Abstract

Sensory neuron types have been distinguished by distinct morphological and transcriptional characteristics. Excitability is the most fundamental functional feature of neurons. Mathematical models described by Hodgkin have revealed three types of neuronal excitability based on the relationship between firing frequency and applied current intensity. However, whether natural sensory neurons display different functional characteristics in terms of excitability and whether this excitability type undergoes plastic changes under pathological pain states have remained elusive. Here, by utilizing whole-cell patch clamp recordings, behavioral and pharmacological assays, we demonstrated that large dorsal root ganglion (DRG) neurons can be classified into three classes and four subclasses based on their excitability patterns, which is similar to mathematical models raised by Hodgkin. Analysis of hyperpolarization-activated cation current (*I*_h_) revealed different magnitude of *I*_h_ in different excitability types of large DRG neurons, with higher *I*_h_ in Class 2-1 than that in Class 1, 2-2 and 3. This indicates a crucial role of *I*_h_ in the determination of excitability type of large DRG neurons. More importantly, this pattern of excitability displays plastic changes and transition under pathological pain states caused by peripheral nerve injury. This study sheds new light on the functional characteristics of large DRG neurons and extends functional classification of large DRG neurons by integration of transcriptomic and morphological characteristics.

## 1. Introduction

Dorsal root ganglion (DRG) neurons are known as pseudo-unipolar sensory neurons, which play crucial roles in transmitting sensory signals from the periphery to the spinal cord. Amongst them, small diameter DRG neurons, mainly consisting of unmyelinated C-fibers and thinly myelinated Aδ-fibers, transmit nociceptive, thermal and mechanoreceptive signals to neurons in nociceptive lamina (lamina I–II) of the spinal cord. In parallel, large diameter DRG neurons convey nonnociceptive signals from the periphery to lamina III–V of the spinal cord via myelinated Aβ-fibers [1]. DRG neurons have been classified based on morphological and transcriptomic characteristics [2,3,4,5,6,7]. Morphologically, DRG neurons are categorized into isolectin B4 (IB4)-positive and peptidergic small neurons as well as neurofilament 200-expressing large neurons [2,3,4,6]. More recently, by using high-coverage RNA-seq, DRG neurons were classified into 10 types and 14 subordinate subtypes with distinct transcriptional patterns [5]. Despite morphological and transcriptomic classification, whether these neurons display different functional characteristics has remained elusive.

Excitability is the most fundamental functional feature of neurons. It not only manifests as different threshold and frequency, but also as different patterns. For example, Hodgkin’s pioneering work with mathematical models has revealed three types of neuronal excitability based on the relationship between firing frequency and applied current intensity [8]. The action potentials of Class 1 excitability are sensitive to the strength of any applied current and are of arbitrarily low frequency. Those of Class 2 are generated in a definite frequency band that is relatively insensitive to applied current strength. For Class 3 excitability, a single action potential is usually generated in response to current pulses at different intensities, with three to five spikes generated only when extremely strong currents are injected [9]. In support of mathematical models, different types of excitability has been observed in natural cells, including neurons in the rat mesencephalic V and supratrigeminal regions, as well as somatosensory cortex [10,11,12,13]. Compelling evidence from animal models has shown that large DRG neurons play a crucial role in mediating neuropathic pain caused by nerve injury [14,15,16,17,18,19,20,21,22,23]. However, little is known about the functional classification of large DRG neurons, and whether their excitability patterns are altered under pathological states. If so, what mechanisms are involved in this process?

To address the above questions, we used a combination of whole-cell patch clamp recordings, behavioral surveys as well as pharmacological assays to investigate the functional classification of large DRG neurons. In the present study, we demonstrated that large DRG neurons can be classified into three classes and four subclasses based on their excitability patterns, which is similar to mathematical models raised by Hodgkin. Analysis of hyperpolarization-activated cation current (*I*_h_) revealed different magnitude of *I*_h_ in different excitability types of large DRG neurons, with higher *I*_h_ in Class 2-1 than that in Class 1, 2-2 and 3. This indicates a crucial role of *I*_h_ in the determination of excitability type of large DRG neurons. More importantly, this pattern of excitability display plastic changes and transition under neuropathic pain states caused by peripheral nerve injury. This study sheds new light on the functional characteristics of large DRG neurons and extends the functional classification of large DRG neurons by integration of transcriptomic, morphological characteristics.

## 2. Results

**Figure 1:**
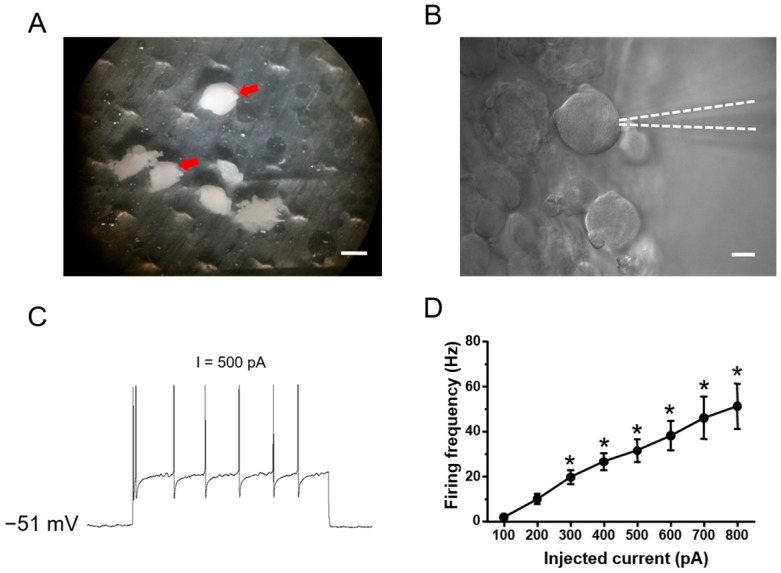

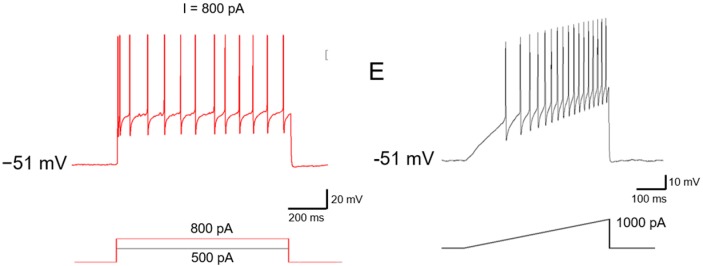
(**A**) Showing intact whole mount dorsal root ganglion (DRG) preparations (red arrow: whole mount DRG, scale bar: 2 mm); (**B**) Showing large-diameter DRG neurons from an intact whole mount L4/5 DRG preparation are recorded (white dash line: electrode, scale bar: 25 μm); (**C**) Current steps were injected into the patched somas through recording electrodes and are shown below the corresponding recording traces. A representative Class 1 neuron is shown. At resting membrane potential (RMP) of −51 mV, the cell responded to intracellular current steps with repetitive action potential firing; (**D**) Analysis of intensity-frequency (I-F) relationship revealed that the firing frequency is approximately linear with respect to current intensities (*n* = 7, Uncorrected Fisher’s LSD one-way ANOVA, F(7,41) = 11.05, *p* < 0.0001; in multiple comparisons, * *p* < 0.05 vs. 100 pA injection); (**E**) A similar dependency of spike frequency on current intensities was confirmed in this subgroup of neurons when current steps were replaced by current ramps. A depolarizing current ramp (1000 pA) caused the neurons to fire spikes at continuously increasing frequency (*n* = 7). Note that (**C**,**E**) are recorded from the same neuron.

Whole-cell patch-clamp recordings were obtained from 242 large DRG neurons with diameter >50 µm in whole mount DRG preparations (Figure 1A,B). Unlike the abnormal hyperexcitability in acutely dissociated or cultured DRG neuron preparation, DRG neurons in whole mount preparation remain intact and close to physiological condition [24]. Neurons were selected for recording if they had a resting membrane potential negative than −50 mV. Almost none of the cells tested showed spontaneous action potentials under resting conditions, while the vast majority showed overshooting action potentials in response to somatic injection of depolarizing current steps.

### 2.1. Functional Classification of Large DRG Neurons under Physiological States

Large DRG neurons were recorded under current-clamped mode. Spike discharges were induced by injection of depolarizing current steps or ramps via recording electrodes. Based on the relationship between firing frequency and applied current intensity, large DRG neurons can be classified into three different classes and four subclasses. This is consistent with experimental observations [8] and the predications of modeling studies [25,26].

Class 1 neurons: In comparison with other classes, Class 1 neurons is more sensitive to depolarizing current injections, manifesting as a lower rheobase at 50–200 pA in response to depolarizing current steps. With the increase of intensity of depolarizing current injection, this subgroup of neurons discharges more spikes (see Figure 1C for typical examples). Furthermore, analysis of intensity-frequency (I-F) relationship revealed that the firing frequency is approximately linear with respect to current intensities (Figure 1D for quantitative summary, *n* = 7). It also holds true when current steps were replaced with current ramps. As shown in Figure 1E, a depolarizing current ramp (1000 pA) caused the neurons to fire spikes at continuously increasing frequency (Figure 1E for typical examples). Class 1 neurons account for about 12.8% of all the neurons examined (*n* = 23 out of 179 neurons tested from five to eight animals).

**Figure 2:**
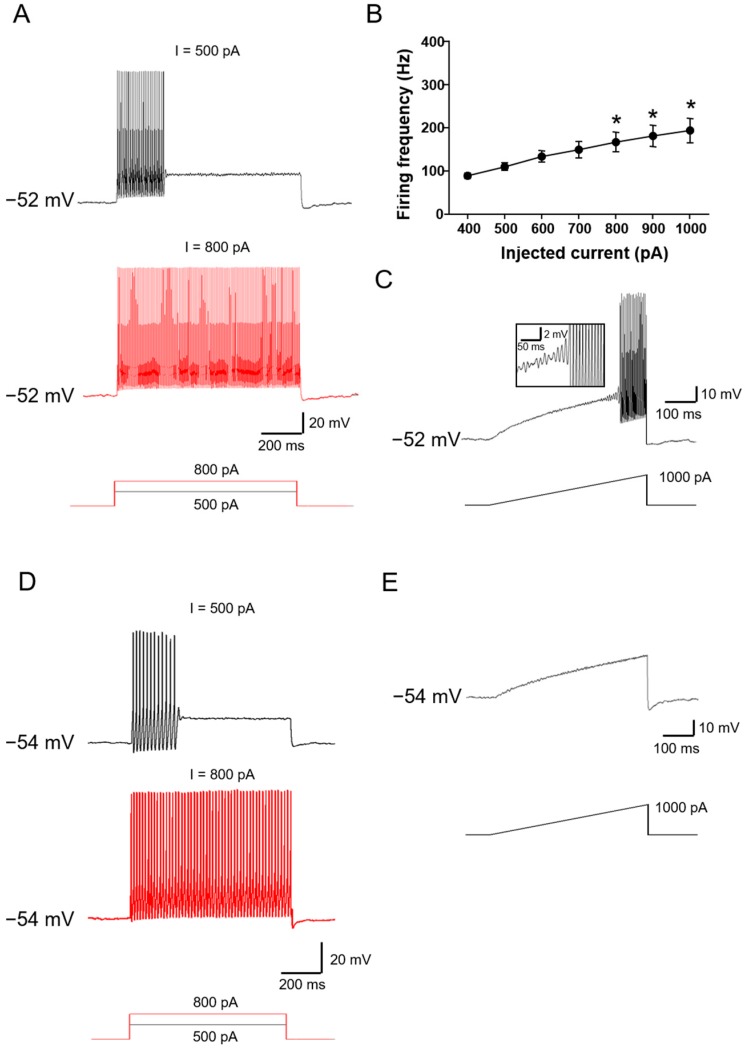
Characteristics of Class 2 large DRG neurons. (**A**–**C**) showing a representative example of Class 2-1 neuron. (**A**) Current steps were injected into the patched somas through recording electrodes and are shown below the corresponding recording traces. At an RMP of −52 mV, when injected with increasing intensity of depolarizing current steps, this group of neurons fires more action potentials, but at a constant frequency (*n* = 8); (**B**) I-F curve revealed that the firing frequency in this class of neurons is independent on stimulus intensity applied (Uncorrected Fisher’s LSD one-way ANOVA, F(6,45) = 4.411, *p* = 0.0014; in multiple comparisons, * *p* < 0.05 vs. 400 pA injection); (**C**) Current ramps were injected into the cell and induced repetitive action potential firing. This subclass of Class 2 neurons (Class 2-1) displayed rhythmical bursting with subthreshold membrane potential oscillations in response to current ramps. Insets show magnified subthreshold membrane potential oscillations. Note that (**A**,**C**) are recorded from the same neuron (*n* = 17); (**D**,**E**) A representative example of Class 2-2 neuron; (**D**) Repetitive action potential firings in response to different current steps shown below; (**E**) Current ramp injection did not induce any action potential firing in this subclass of neurons (*n* = 7).

Class 2 neurons: As compared to Class 1 neurons, Class 2 neurons showed a higher rheobase at 200–500 pA in response to depolarizing current steps. When injected with increasing intensity of depolarizing current steps, this group of neurons fires more action potentials, but at a constant frequency (see Figure 2A for typical examples). I-F curve revealed that the firing frequency in this class of neurons is independent on stimulus intensity applied (see Figure 2B for quantitative summary). 26.8% of the neurons fall into Class 2 type (*n* = 48 out of 179 neurons tested from five to eight animals). Apart from depolarizing current steps, current ramps were also applied to confirm this functional characteristic. Interestingly, two different responses to current ramps were observed. 70.8% of Class 2 neurons displayed rhythmical bursting with subthreshold membrane potential oscillations in response to current ramps, which is consistent with what is observed in mesencephalic V neurons [13] (Figure 2C). We named this subclass of neurons Class 2-1. In parallel, 29.2% of Class 2 neurons were not responding to ramp currents at all, even without subthreshold membrane potential oscillations, named Class 2-2 (Figure 2D).

**Figure 3:**
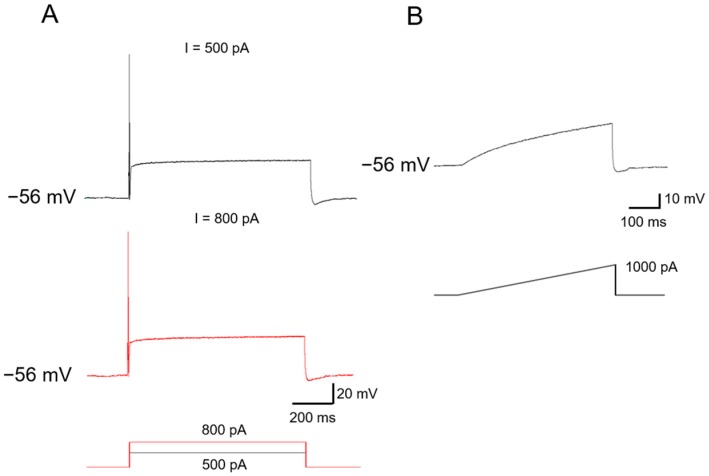
Typical examples of Class 3 neurons. (**A**) Typical recording traces showing that only one spike can be induced by depolarizing current steps (*n* = 17); (**B**) In this class of neurons, no action potential was induced by current ramp injections. Note (**A**,**B**) were recorded from the same neuron.

Class 3 neurons: Class 3 neurons are rather dull in relation to depolarizing current injections, showing the highest rheobase (>500 pA) to evoke action potential. The most remarkable feature of Class 3 neurons is that only one spike can be induced by depolarizing current steps (Figure 3A). Even when the intensity of current injections is increased as high as 2 nA, no more than three to five spikes were evoked in a few neurons. Furthermore, a current ramp as high as 1000 pA failed to evoke any response (Figure 3B). Class 3 neurons account for about 60.3% of all the neurons examined (*n* = 108 out of 179 neurons tested from five to eight animals).

To further characterize these difference classes of DRG neurons, a series of passive membrane properties were examined. As shown in Table 1, the passive membrane properties of three classes and four subclasses of DRG neurons were similar (Table 1). None of resting membrane potential (RMP), membrane capacitance and resistance in different subclasses of large DRG neurons were different (Table 1, Uncorrected Fisher’s LSD one-way ANOVA, RMP: F(3,35) = 2.000, *p* = 0.1318; Cm: F(3,25) = 0.8018, *p* = 0.5046; Rm: F(3,25) = 0.3494, *p* = 0.7899).

### 2.2. Ionic Mechanisms Underlying Different Excitability Type of Large DRG Neurons

**Figure 4:**
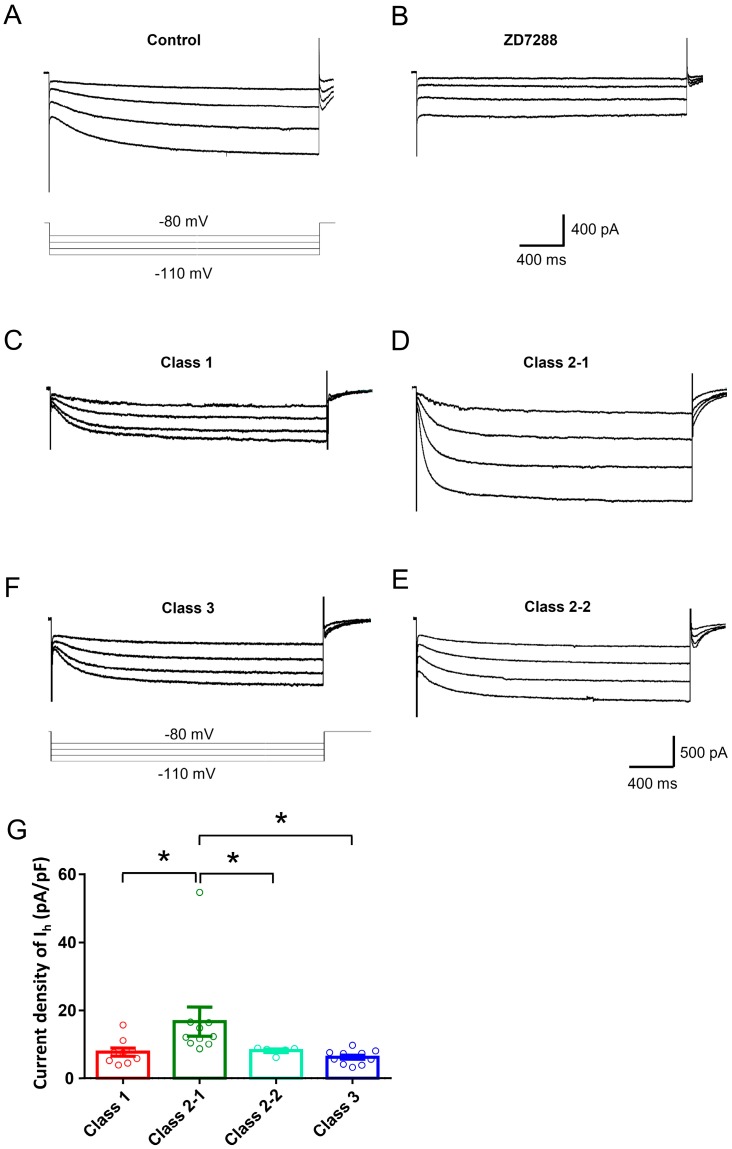
(**A**,**B**) Representative traces of *I*_h_ recorded in large DRG neurons in response to hyperpolarizing voltage steps of −60 to −120 mV from a holding potential at −60 mV (**A**) and its blockade by ZD7288 (15 µM); (**C**–**F**) Representative traces of *I*_h_ recorded in different classes of large DRG neurons; (**G**) Analyses of current density of *I*_h_ revealed that the magnitude of *I*_h_ in Class 2-1 neurons was much higher than that in Class 1, 3 and 2-2 neurons (*n* = 5–11, Uncorrected Fisher’s LSD one-way ANOVA, F(3,31) = 3.869, *p* = 0.0185, and in multiple comparisons * *p* = 0.0151 for Class 2-1 vs. Class 1, * *p* = 0.0469 for Class 2-1 vs. Class 2-2, * *p* = 0.0036 for Class 2-1 vs. Class 3).

Much evidence has accumulated showing that hyperpolarization-activated cation current (*I*_h_) is important in influencing the excitability and driving repetitive firing in primary nociceptive neurons and pain pathophysiology [27,28,29]. An important role of persistent sodium current (*I*_Na,P_) and potassium current has been revealed in the determination of excitability type reported in mesencephalic V neurons [13]. In the present study, we are interested to know whether *I*_h_ is important in determining different types of excitability in large DRG neurons. Consistent with previous reports [30,31,32], *I*_h_ recorded in our study displays as an inward current in response to hyperpolarizing potentials of −60 to −120 mV from a holding potential of −60 mV (Figure 4A–D for typical examples). This inward current is highly sensitive to ZD7288 (15 µM), a selective blocker of *I*_h_ [32] (Figure 4A,B). Although inward rectifier K^+^ current could also be evoked by these hyperpolarizing voltage steps, this current is unlikely to contribute to the recorded current because of its fast, instantaneous activation kinetics and very low expression in DRG neurons [33].

Quantitative analysis revealed that amongst four subclasses DRG neurons, the magnitude of *I*_h_ in Class 2-1 neurons was much higher than that in Class 1, 3 and 2-2 neurons (Figure 4C–F for typical examples and Figure 4E for quantitative summary, Uncorrected Fisher’s LSD one-way ANOVA, F(3,31) = 3.869, *p* = 0.0185,, *n* = 5–11). The magnitude of *I*_h_ in Class 3 neurons was lowest in four different subclasses. Class 1 neurons has slightly higher amplitude of *I*_h_ than that of Class 3, but it did not reach statistical significance (Figure 4G, *p* = 0.6655, *n* = 5–11). Together with firing properties to injected current injection, these results indicate that *I*_h_ expression is closely related with different types of excitability in large DRG neurons.

### 2.3. Development of Mechanical Hypersensitivity and Thermal Hyperalgesia in Rats Subjected to Chronic Compression of Lumbar DRG

**Figure 5:**
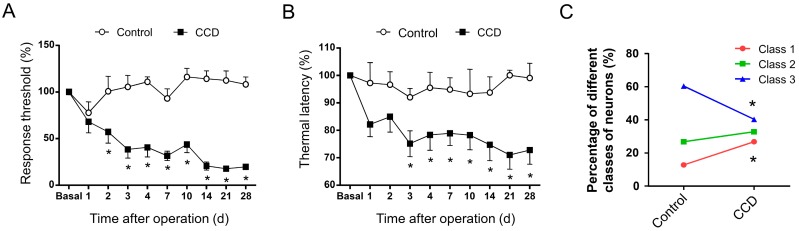
(**A**,**B**) Development of mechanical hypersensitivity and thermal hyperalgesia following chronic compression of DRG (CCD) in rats; (**A**) Mechanical hypersensitivity developed in the ipsilateral hind paws from CCD rats but not in sham controls. Note that paw withdrawal mechanical threshold (PWMT) to von Frey hairs decreased dramatically from the second day following chronic compression of L4/L5 DRG (*n* = 10; Two way ANOVA, F(1,150) = 218.5, *p* < 0.0001; in multiple comparisons, * *p* < 0.05 vs. Control); (**B**) Thermal hyperalgesia was observed by measuring paw withdrawal thermal latency (PWTL) to radiant heat in CCD rats and sham controls for ipsilateral hind paws. Note that PWTL significantly decreased in CCD rats (*n* = 10; Two way ANOVA, F(1,140) = 41.67, *p* < 0.0001; in multiple comparisons, * *p* < 0.05 vs. Control), but not in sham group with a similar time course with mechanical hypersensitivity; (**C**) Upon chronic compression of lumbar DRGs, the percentage of neurons showing different classes of excitability underwent plastic changes. The proportion of Class 1 and Class 3 neurons reciprocally displayed marked increase and decrease (Class 1: Pearson χ^2^ test, F = 9.842, * *p* = 0.002; Class 3: Pearson χ^2^ test, F = 12.321, * *p* < 0.001). Class 2 neurons did not show obvious alteration (*n* = 22–48, Pearson χ^2^ test, F = 1.338, *p* = 0.247). All data are expressed as mean ± S.E.M.

Following chronic compression of L4/L5 DRG (CCD), the rats appeared in good health and did not show any signs of autotomy throughout the study. The sensitivity of CCD rats to mechanical and thermal stimuli was tested at different time points post operation. Compared to sham controls, CCD rats developed dramatic mechanical hypersensitivity (allodynia and hyperalgesia), manifesting as a significant decrease in response threshold to von Frey hairs application to the injured hind paws (Figure 5A, *n* = 10, *p* < 0.05). This mechanical hypersensitivity appeared on the second day after compression, persisting over the entire experimental period. In parallel, a dramatic drop in response latency to noxious plantar heat stimuli, reflecting thermal hyperalgesia, was found in ipsilateral hind paws of CCD rats (Figure 5B, *n* = 10, *p* < 0.05). Therefore, it can be inferred that rats with chronic compression of L4/L5 DRGs develop strong mechanical hypersensitivity and thermal hyperalgesia, which is consistent with previous reports in rodents [13,15,18].

### 2.4. Chronic Compression of Lumbar DRGs Induces Transition of Excitability Type in Large DRG Neurons

To address whether the excitability type of large DRG neurons in physiological states undergoes plastic changes in chronic pain states, we performed chronic compression of L4/L5 DRGs to mimic low back pain symptoms in clinic. Totally, we compared the excitability type of 179 and 134 neurons from five to eight rats in control and CCD group, respectively. Similar to control DRG neurons, three classes of excitability were observed in injured DRG neurons (Figure 5C). However, the percentage of neurons showing different classes of excitability underwent plastic changes. Following chronic compression of lumbar DRGs, the percentage of Class 1 neurons was increased from 12.8% to 26.9% (Figure 5C, *p* < 0.05, *n* = 36 out of 134 neurons tested from five to eight CCD rats). In striking contrast, Class 3 neurons displayed dramatic reduction in quantity from 60.3% to 40.3% (Figure 5C, *p* < 0.05, *n* = 54 out of 134 neurons tested from five to eight CCD rats). Unlike marked changes in Class 1 and 3 neurons, the percentage of Class 2 neuron did not show obvious alteration after chronic compression of lumbar DRGs (Figure 5C, *p* > 0.05, *n* = 44 out of 134 neurons tested from five to eight CCD rats). Considering the lower threshold of Class 1 neurons versus Class 3 neurons, this dramatic increase of Class 1 and decrease of Class 3 in injured DRG neurons is consistent with the reported enhancement of excitability after CCD operation [14,18].

## 3. Discussion

Mathematical models described by Hodgkin have revealed three types of neuronal excitability based on the relationship between firing frequency and applied current intensity [8]. Our present study provided direct evidence in experimental conditions that large DRG sensory neurons can be classified into three classes in terms of excitability patterns. More importantly, this excitability patterns can be dynamically changed under pathological states. Further studies indicated that *I*_h_ are critical determinants in the excitability patterns of large DRG neurons.

Neuron types have often been defined by their morphological characteristics. For example, depending on the binding of isolectin B4, small-diameter DRG neurons are categorized into IB4-positive nonpeptidergic and IB4-negative peptidergic neurons, while large DRG neurons are usually NF200-positive [2,3,4,6]. Recently, multiple efforts have been made to analyze the transcriptional profiles of DRG neurons [34,35,36,37]. More recently, by integrating single-cell techniques such as high-coverage RNA-seq, in vivo patch clamp recording and single-cell PCR, Li et al. classified DRG neurons into 10 types and 14 subordinate subtypes with distinct transcriptional patterns, molecular markers and functional annotations [5]. However, neuron type-specific functional analyses are needed to confirm and elaborate the precise functions of these neuron types. Excitability is the most fundamental and important functional features of neurons. Traditionally, different excitability is assumed to be based on firing frequency, namely higher excitability with higher firing frequency and vice versa. In 1948, Hodgkin put forward a theory about classification of excitability in terms of firing patterns based on mathematical models [8]. Whether natural DRG neurons display different excitability types based on firing patterns and whether this excitability type undergoes plastic changes under pathological states have remained elusive.

One of most striking findings of the present study is that we classified large DRG neurons into three classes and four subclasses based on the excitability and firing patterns. Class 1 neurons fire at low threshold, and its firing rate are linearly correlated with the intensity of injected current. Class 2 neurons fire at a much higher rate, which is not dependent on the intensity of current injected. Class 3 neurons show only single or a few spikes even at very high current injection. Depending on the response to ramp current, Class 2 neurons were further classified into two subclasses, one with subthreshold membrane oscillation and high frequency firing and one with no response at all in response to ramp current. In a recent study by Li et al. [5], large DRG neurons were classified into four clusters and six subclusters in a transcriptional profile. The present study extends this transcriptional classification of large DRG neurons in a functional basis. However, how this functional classification corresponds to transcriptional and morphological classification remains to be further determined.

Several ionic currents have been implicated in the membrane excitability and repetitive firing in neurons including *I*_NaP_, *I*_h_ [15,38,39,40]. Previous studies in mesencephalic V neurons have reported a crucial role of *I*_Na,P_ in the determination of excitability type [13]. In the present study, however, we demonstrated for the first time that *I*_h_ is important in determining different types of excitability in large DRG neurons. Amongst four subclasses of excitability of large DRG neurons, Class 2-1 neurons displayed the highest magnitude of *I*_h_, while Class 3 neurons exhibited the lowest magnitude of *I*_h_. Given the firing patterns in response to injected current in different types of neurons, it indicates that *I*_h_ is closely related with different types of excitability in large DRG neurons. This is consistent with previous studies showing that *I*_h_ is important in driving repetitive firing and influencing excitability of DRG neurons and pain hypersensitivity [27,28,29,31,32,39,41].

Another striking finding of the present study is that neuronal excitability type is not static, but undergoes dynamic changes under chronic pain states. Following chronic compression of lumbar DRGs, proportion of different excitability types of large DRG neurons displayed an obvious alteration. As compared to control group, chronic compression of lumbar DRGs produced a dramatic increase of Class 1 neurons and a marked decrease of Class 3 neurons reciprocally. In contrast, the proportion of Class 2 neurons did not show obvious alteration. This change indicates some Class 3 neurons may transit into Class 1 under pathological states. Considering the lower threshold and high firing frequency of Class 1 neurons, this transition in excitability type might reflect the enhanced neuronal excitability reported previously in large DRG neurons under chronic pain states [15,18,24,38].

Taken together, our results show that the excitability of large DRG neurons can be classified into three classes and four subclasses in terms of firing patterns in response to injected current. Hyperpolarization-activated cation currents are crucial in determination of different types of excitability. This excitability type of large DRG neurons underwent a plastic changes in chronic pain states.

## 4. Methods

### 4.1. Animals

Experiments were performed on adult, male Sprague-Dawley rats (100–150 g). The rats were housed in an accredited animal facility room controlled for temperature (23.5 ± 0.7 °C), humidity (34.0 ± 5.7%), and light (12-h light-dark cycle). The animals were fed a standard chow and water ad libitum. All experimental protocols were approved by an institutional animal use and protection committee. The Foutrh Military Medical University Animal Centre ethics committee (20150102, 15 January 2015).

### 4.2. Chronic Compression of Lumbar DRGs (CCD)

The methods for CCD operation have been described in detailed elsewhere [14,15,42]. In brief, the skin was incised on the left side of lumbar vertebrae between L3 and L6 and paraspinal muscles were separated from vertebrae at the L3–L6 level to expose the L4 and L5 intervertebral foramen clearly. A fine, L-shaped needle was inserted into the L4 and L5 intervertebral foramen at a 30° angle with respect to the dorsal middle line and a 20° angle with respect to the vertebral horizontal line. After the rods were in place, the muscle and skin layers were sutured with administration of about 200 mg antibiotics to prevent infections. Three to five days later, when stable hyperalgesia and allodynia was established, injured DRGs were dissected and subjected to further recording.

### 4.3. Behavioral Tests

Behavioral testing was carried out in habituated rat by an observer blinded to the identity of the groups. As previously described [43,44], mechanical sensitivity was tested with manual application of Von Frey hairs (North Coast) to the plantar surface of the hind paw. Each filament was applied 10 times and the paw withdrawal response frequency (the percentage of positive responses to the stimulus) was recorded. The force of a particular filament required to elicit 50% frequency of paw withdrawal was expressed as the mechanical threshold. Thermal sensitivity was tested by application of infrared heat to the plantar surface of the hind paw and the response latency was measured from an automated device readout (IITC Life Science, Woodlands Hills, CA, USA).

### 4.4. Intact Whole Mount DRG Preparations

The procedure for an ex vivo preparation of intact whole mount DRG has been described elsewhere [15,38,43]. In brief, L4 and L5 DRGs were carefully removed at postoperative 3–5 day from CCD rats or sham control rats. After removing the connective tissue, the ganglia were digested with a mixture of 0.4 mg/mL trypsin (Sigma, St. Louis, MO, USA) and 1.0 mg/mL type-A collagenase (Sigma) for 45 min at 37 °C. The intact ganglia were then incubated in artificial cerebrospinal fluid (ACSF) oxygenated with 95% O_2_ and 5% CO_2_ at 28 °C for at least 1 h before transferring them to the recording chamber.

### 4.5. Whole Cell Patch-Clamp Recording

DRG neurons were visualized with a 40× water-immersion objective using a microscope (BX51WI; Olympus, Tokyo, Japan) equipped with infrared differential interference contrast optics. Whole-cell current and voltage recordings were acquired with an Axon700B amplifier (Molecular Devices Corporation, Sunnyvale, CA, USA). Patch pipettes (4–7 MΩ) were pulled from borosilicate glass capillaries on P-97 puller (Sutter Instruments, San Rafael, CA, USA). The series resistance was 10–20 MΩ. Neurons were selected for further study if they had a resting membrane potential negative than −50 mV and if they exhibited overshooting action potentials.

The large-diameter L4 and L5 DRG neurons (>50 µm) were recorded. The ACSF contained (in mM): 124 NaCl, 2.5 KCl, 1.2 NaH_2_PO_4_, 1.0 MgCl_2_, 2.0 CaCl_2_, 25 NaHCO_3_, and 10 Glucose. The pipette solution contained (in mM): 140 KCl, 2 MgCl_2_, 10 Hepes, 2 Mg-ATP, pH 7.4. Osmolarity was adjusted to 290–300 mOsm. All chemicals were obtained from Sigma. Data was acquired with a digidata 1322A acquisition system (molecular devices) using pCLAMP 9.0 software. Signals were low-pass filtered at 5 kHz, sampled at 10 kHz and analyzed offline.

### 4.6. I_h_ Current Analysis

Recording of *I*_h_ was as described previously [39]. Briefly, for recording of *I*_h_, ACSF and normal intrapipette solution mentioned above were used. To activate *I*_h_, hyperpolarizing potentials of −60 to −20 mV were delivered in increments of 10 mV from a holding potential of −60 mV for duration of 2.5 s. For each neuron, *I*_h_ was measured as the difference between the steady state current and the initial (instantaneous) inward current. The initial *I*_h_ was measured 12 to 15 ms after the start of the voltage command to avoid contamination by the capacitance artifacts [45].

### 4.7. Statistical Analysis

All data are expressed as mean ± S.E.M. Student’s *t*-test or an analysis of variance (ANOVA) for random measures was carried out, followed by either a post hoc Fisher’s test or Dunnett’s test. In some cases, two-way ANOVA for repeated measures was conducted. *p* < 0.05 was considered significant.

## Figures and Tables

**Table 1:** Passive membrane properties of different types of large DRG neurons.

Type	RMP (mV)	Cm (GOhms)	Rm (MOhms)
Class 1	−55.38 ± 2.05	102.67 ± 15.54	0.088 ± 0.02
Class 2-1	−57.64 ± 2.25	97.23 ± 10.55	0.099 ± 0.02
Class 2-2	−53.98 ± 1.13	79.69 ± 2.48	0.071 ± 0.02
Class 3	−59.61 ± 1.11	92.36 ± 7.41	0.079 ± 0.01

## Abbreviations

*I*_h_	Hyperpolarization-Activated Cation Currents
DRG	Dorsal Root Ganglion
CCD	Chronic Compression of Dorsal Root Ganglion
IB4	Isolectin B4
*I*_Na,P_	Persistent Sodium Current

## References

[1] Maxwell D.J., Réthelyi M. (1987). Ultrastructure and synaptic connections of cutaneous afferent fibres in the spinal cord. Trends Neurosci..

[2] Li L., Rutlin M., Abraira V.E., Cassidy C., Kus L., Gong S., Jankowski M.P., Luo W., Heintz N., Koerber H.R. (2011). The functional organization of cutaneous low-threshold mechanosensory neurons. Cell.

[3] Ju G., Hokfelt T., Brodin E., Fahrenkrug J., Fischer J.A., Frey P., Elde R.P., Brown J.C. (1987). Primary sensory neurons of the rat showing calcitonin gene-related peptide immunoreactivity and their relation to substance P-, somatostatin-, galanin-, vasoactive intestinal polypeptide- and cholecystokinin-immunoreactive ganglion cells. Cell Tissue Res..

[4] Wang H., Rivero-Melian C., Robertson B., Grant G. (1994). Transganglionic transport and binding of the isolectin B4 from Griffonia simplicifolia I in rat primary sensory neurons. Neuroscience.

[5] Li C.L., Li K.C., Wu D., Chen Y., Luo H., Zhao J.R., Wang S.S., Sun M.M., Lu Y.J., Zhong Y.Q. (2016). Somatosensory neuron types identified by high-coverage single-cell RNA-sequencing and functional heterogeneity. Cell Res..

[6] Meyer R.A., Ringkamp M., Campbell J., Raja S. (2006). Peripheral mechanisms of cutaneous nociception. Wall & Melzack’s Textbook of Pain.

[7] Li C., Wang S., Chen Y., Zhang X. (2017). Somatosensory Neuron Typing with High-Coverage Single-Cell RNA Sequencing and Functional Analysis. Neurosci. Bull..

[8] Hodgkin A.L. (1948). The local electric changes associated with repetitive action in a non-medullated axon. J. Physiol..

[9] Izhikevich E.M. (2007). Dynamical Systems in Neuroscience: The Geometry of Excitability and Bursting.

[10] Hsiao C.F., Gougar K., Asai J., Chandler S.H. (2007). Intrinsic membrane properties and morphological characteristics of interneurons in the rat supratrigeminal region. J. Neurosci. Res..

[11] Hsiao C.F., Kaur G., Vong A., Bawa H., Chandler S.H. (2009). Participation of Kv1 channels in control of membrane excitability and burst generation in mesencephalic V neurons. J. Neurophysiol..

[12] Tateno T., Harsch A., Robinson H.P. (2004). Threshold firing frequency-current relationships of neurons in rat somatosensory cortex: Type 1 and type 2 dynamics. J. Neurophysiol..

[13] Yang J., Xing J.L., Wu N.P., Liu Y.H., Zhang C.Z., Kuang F., Han V.Z., Hu S.J. (2009). Membrane current-based mechanisms for excitability transitions in neurons of the rat mesencephalic trigeminal nuclei. Neuroscience.

[14] Hu S.J., Xing J.L. (1998). An experimental model for chronic compression of dorsal root ganglion produced by intervertebral foramen stenosis in the rat. Pain.

[15] Song Y., Li H.M., Xie R.G., Yue Z.F., Song X.J., Hu S.J., Xing J.L. (2012). Evoked bursting in injured Abeta dorsal root ganglion neurons: A mechanism underlying tactile allodynia. Pain.

[16] Kim Y.I., Na H.S., Kim S.H., Han H.C., Yoon Y.W., Sung B., Nam H.J., Shin S.L., Hong S.K. (1998). Cell type-specific changes of the membrane properties of peripherally-axotomized dorsal root ganglion neurons in a rat model of neuropathic pain. Neuroscience.

[17] Liu C.N., Wall P.D., Ben-Dor E., Michaelis M., Amir R., Devor M. (2000). Tactile allodynia in the absence of C-fiber activation: Altered firing properties of DRG neurons following spinal nerve injury. Pain.

[18] Zhang J.M., Song X.J., LaMotte R.H. (1999). Enhanced excitability of sensory neurons in rats with cutaneous hyperalgesia produced by chronic compression of the dorsal root ganglion. J. Neurophysiol..

[19] Zhu Y.F., Henry J.L. (2012). Excitability of Abeta sensory neurons is altered in an animal model of peripheral neuropathy. BMC Neurosci..

[20] Jin S.X., Zhuang Z.Y., Woolf C.J., Ji R.R. (2003). p38 mitogen-activated protein kinase is activated after a spinal nerve ligation in spinal cord microglia and dorsal root ganglion neurons and contributes to the generation of neuropathic pain. J. Neurosci..

[21] Liu C.N., Michaelis M., Amir R., Devor M. (2000). Spinal nerve injury enhances subthreshold membrane potential oscillations in DRG neurons: Relation to neuropathic pain. J. Neurophysiol..

[22] Xiao H.S., Huang Q.H., Zhang F.X., Bao L., Lu Y.J., Guo C., Yang L., Huang W.J., Fu G., Xu S.H. (2002). Identification of gene expression profile of dorsal root ganglion in the rat peripheral axotomy model of neuropathic pain. Proc. Natl. Acad. Sci. USA.

[23] Dib-Hajj S.D., Fjell J., Cummins T.R., Zheng Z., Fried K., LaMotte R., Black J.A., Waxman S.G. (1999). Plasticity of sodium channel expression in DRG neurons in the chronic constriction injury model of neuropathic pain. Pain.

[24] Zheng J.H., Walters E.T., Song X.J. (2007). Dissociation of dorsal root ganglion neurons induces hyperexcitability that is maintained by increased responsiveness to cAMP and cGMP. J. Neurophysiol..

[25] Izhikevich E.M. (2000). Neural excitability, spiking and bursting. Int. J. Bifurc. Chaos.

[26] Rinzel J., Ermentrout G.B., Christof K., Idan S. (1989). Analysis of neural excitability and oscillations. Methods in Neuronal Modeling.

[27] Chaplan S.R., Guo H.Q., Lee D.H., Luo L., Liu C., Kuei C., Velumian A.A., Butler M.P., Brown S.M., Dubin A.E. (2003). Neuronal hyperpolarization-activated pacemaker channels drive neuropathic pain. J. Neurosci..

[28] Luo L., Chang L., Brown S.M., Ao H., Lee D.H., Higuera E.S., Dubin A.E., Chaplan S.R. (2007). Role of peripheral hyperpolarization-activated cyclic nucleotide-modulated channel pacemaker channels in acute and chronic pain models in the rat. Neuroscience.

[29] Emery E.C., Young G.T., Berrocoso E.M., Chen L., McNaughton P.A. (2011). HCN2 ion channels play a central role in inflammatory and neuropathic pain. Science.

[30] Biel M., Wahl-Schott C., Michalakis S., Zong X. (2009). Hyperpolarization-activated cation channels: From genes to function. Physiol. Rev..

[31] Weng X., Smith T., Sathish J., Djouhri L. (2012). Chronic inflammatory pain is associated with increased excitability and hyperpolarization-activated current *I*_h_ in C- but not Adelta-nociceptors. Pain.

[32] Liu D.-L., Wang X., Chu W.-G., Lu N., Han W.-J., Du Y.-K., Hu S.-J., Bai Z.-T., Wu S.-X., Xie R.-G. (2017). Chronic cervical radiculopathic pain is associated with increased excitability and hyperpolarization-activated current (*I*_h_) in large-diameter dorsal root ganglion neurons. Mol. Pain.

[33] Scroggs R.S., Todorovic S.M., Anderson E.G., Fox A.P. (1994). Variation in IH, IIR, and ILEAK between acutely isolated adult rat dorsal root ganglion neurons of different size. J. Neurophysiol..

[34] Goswami S.C., Mishra S.K., Maric D., Kaszas K., Gonnella G.L., Clokie S.J., Kominsky H.D., Gross J.R., Keller J.M., Mannes A.J. (2014). Molecular signatures of mouse TRPV1-lineage neurons revealed by RNA-Seq transcriptome analysis. J. Pain.

[35] Thakur M., Crow M., Richards N., Davey G.I., Levine E., Kelleher J.H., Agley C.C., Denk F., Harridge S.D., McMahon S.B. (2014). Defining the nociceptor transcriptome. Front. Mol. Neurosci..

[36] Chiu I.M., Barrett L.B., Williams E.K., Strochlic D.E., Lee S., Weyer A.D., Lou S., Bryman G.S., Roberson D.P., Ghasemlou N. (2014). Transcriptional profiling at whole population and single cell levels reveals somatosensory neuron molecular diversity. eLife.

[37] Usoskin D., Furlan A., Islam S., Abdo H., Lonnerberg P., Lou D., Hjerling-Leffler J., Haeggstrom J., Kharchenko O., Kharchenko P.V. (2015). Unbiased classification of sensory neuron types by large-scale single-cell RNA sequencing. Nat. Neurosci..

[38] Yang R.H., Wang W.T., Chen J.Y., Xie R.G., Hu S.J. (2009). Gabapentin selectively reduces persistent sodium current in injured type-A dorsal root ganglion neurons. Pain.

[39] Liu D.L., Lu N., Han W.J., Chen R.G., Cong R., Xie R.G., Zhang Y.F., Kong W.W., Hu S.J., Luo C. (2015). Upregulation of *I*_h_ expressed in IB4-negative Adelta nociceptive DRG neurons contributes to mechanical hypersensitivity associated with cervical radiculopathic pain. Sci. Rep..

[40] Yang R.H., Xing J.L., Duan J.H., Hu S.J. (2005). Effects of gabapentin on spontaneous discharges and subthreshold membrane potential oscillation of type A neurons in injured DRG. Pain.

[41] Ingram S.L., Williams J.T. (1996). Modulation of the hyperpolarization-activated current (*I*_h_) by cyclic nucleotides in guinea-pig primary afferent neurons. J. Physiol..

[42] Xie R.G., Zheng D.W., Xing J.L., Zhang X.J., Song Y., Xie Y.B., Kuang F., Dong H., You S.W., Xu H. (2011). Blockade of persistent sodium currents contributes to the riluzole-induced inhibition of spontaneous activity and oscillations in injured DRG neurons. PLoS ONE.

[43] Sun W., Miao B., Wang X.C., Duan J.H., Wang W.T., Kuang F., Xie R.G., Xing J.L., Xu H., Song X.J. (2012). Reduced conduction failure of the main axon of polymodal nociceptive C-fibres contributes to painful diabetic neuropathy in rats. Brain.

[44] Xie R.G., Gao Y.J., Park C.K., Lu N., Luo C., Wang W.T., Wu S.X., Ji R.R. (2017). Spinal CCL2 Promotes Central Sensitization, Long-Term Potentiation, and Inflammatory Pain via CCR2: Further Insights into Molecular, Synaptic, and Cellular Mechanisms. Neurosci. Bull..

[45] Rodrigues A.R., Oertel D. (2006). Hyperpolarization-activated currents regulate excitability in stellate cells of the mammalian ventral cochlear nucleus. J. Neurophysiol..

